# RNA interference core components identified and characterised in *Verticillium nonalfalfae*, a vascular wilt pathogenic plant fungi of hops

**DOI:** 10.1038/s41598-019-44494-8

**Published:** 2019-06-17

**Authors:** Taja Jeseničnik, Nataša Štajner, Sebastjan Radišek, Jernej Jakše

**Affiliations:** 1 University of Ljubljana, Biotechnical Faculty, Agronomy Department, Ljubljana, 1000 Slovenia; 2 Slovenian Institute of Hop Research and Brewing, Žalec, 3310 Slovenia

**Keywords:** Plant immunity, RNAi, Agricultural genetics

## Abstract

The conserved RNA interference mechanism (RNAi) in the fungal kingdom has become a focus of intense scientific investigation. The three catalytic core components, Dicer-like (DCL), Argonaute (AGO), and RNA-dependent RNA polymerase (RdRP), and their associated small interfering RNA molecules (siRNAs) have been identified and characterised in several fungal species. Recent studies have proposed that RNAi is a major contributor to the virulence of fungal pathogens as a result of so-called trans-kingdom RNA silencing. In the present study, we report on the existence of three core RNAi proteins in the pathogenic plant fungus *Verticillium nonalfalfae*, which is a soilborne plant pathogen that causes severe wilting disease in hops (*Humulus lupulus* L.). Two DCL proteins, two AGO proteins, and two RdRP proteins were identified, and their conserved RNAi domains were characterised. Our phylogeny results confirm the existing taxonomic relationships in the Ascomycete fungal phylum and show that the fungi of the Hypocreomycetidae subclass of the Sordariomycetes class have high amino acid sequence similarity. The expression analysis revealed a potential role of RNAi in the pathogenicity of the fungi, since all the RNAi genes were highly upregulated in the highly virulent isolate T2 and were also differentially expressed in the *V. nonalfalfae*-susceptible Celeia and *V. nonalfalfae*-resistant Wye Target cultivars.

## Introduction

The molecular mechanism of RNA interference (RNAi) was first identified in *Caenorhabditis elegans*^1^ and has been described as a negative regulator of gene expression driven by small non-coding RNA molecules (siRNAs). In the fungal model *Neurospora crassa* (*N. crassa*), the phenomenon of transient gene silencing had already been described in 1992 by Romano and Macino^2^, which established a foundation for RNAi discoveries, not only in animals but also in plants^3^ and protists^4^. In the conserved RNAi pathway, the Dicer ribonuclease III enzymes (DCR and DCL in fungi) cleave double-stranded RNA precursors (dsRNAs) to generate short dsRNAs that are subsequently loaded into the RNA-induced silencing complex (RISC), which contains an Argonaute (AGO) protein as the core catalytic component. In the RISC complex, one of the two strands of short dsRNA is removed, and the processed siRNA is complementarily paired with messenger RNA (mRNA), resulting in the inhibition of expression and the silencing of target mRNAs involved in numerous biological processes, such as vegetative development, RNA processing, the maintenance of genome integrity, and immune responses^5–8^. Recently, it was shown that pathogenic plant fungi also use siRNAs as virulence factors by exporting them to the host to mediate host defence, and a trans-kingdom RNA silencing model has been proposed for host-pathogen interactions^9–11^. In fungi, the formation of dsRNA precursors for siRNA synthesis is mediated by RNA-dependent RNA polymerase (RdRP) activity^12,13^.

The first studies of RNAi mechanisms were conducted using *N. crassa* quelling-defective mutants, which led to the identification of the core components of RNAi: RdRP (QDE-1), AGO (QDE-2), and the DNA helicase RecQ (QDE-3)^13,14^. Additionally, two Dicer-like proteins were identified in the *N. crassa* genome (DCL1 and DCL2)^15^. Highly conserved among species, the three core proteins have characteristic structures comprised of several distinct domains. In AGO proteins, the typical structure is based on the N-terminal ArgoN domain followed by the PAZ, MID, and PIWI domains. The DCL proteins contain a DExD-helicase followed by a helicase-C, Dicer-dimerisation domain (a divergent double stranded RNA-binding domain), a PAZ domain, and two RNase III domains. All RdRP proteins contain a catalytic RdRP domain^16^. In recent years, using sequence homology approaches, the DCL, RdRP, and AGO proteins have also been identified in several other fungal species, including *Mucor circinelloides*^17–19^, *Cryptococcus neoformans*^20^, *Penicillium marnefeni*^21^, *Antrodia cinnamomea*^22^, *Fusarium graminearum*^23^, *Metarhizuium robertsii*^24^, and other fungal model organisms^25^. The identification of the core components of RNAi and their associated siRNAs is associated with the advancement and implementation of next-generation sequencing (NGS) technologies^26^, which enables the generation of a large number of sequenced reads for use in the construction of detailed genomes and transcriptomes to allow not only the identification of major proteins and their gene sequences but also of shorter and less abundantly represented RNA molecules in samples and organisms^27^.

*Verticillium* sensu stricto is an anamorphic genus within the pathogenic plant fungi of Ascomycetes that causes verticillium wilt, which is a soil-born vascular disease that affects many important crops worldwide^28,29^. Of the 10 currently described species, *Verticillium dahliae* (*V. dahliae*) is the most widespread fungus and infects more than 200 plant species, whereas other species, such as *V. nonalfalfae*, have a narrower host range but cause significant damage in specific hosts^30^. One of the most susceptible hosts of *V. nonalfalfae* is hop (*Humulus lupulus* L.), which is a dioecious perennial climbing plant primarily cultivated as an essential product for beer production^31^. The most severe outbreaks in hops are caused by highly virulent *V. nonalfalfae* pathotypes that produce infections that induce rapid plant dieback and lethal verticillium wilt symptoms. Hop is also a host of *V. dahliae* and the less virulent strains of *V. nonalfalfae*; infections with these species are less severe and cause mild symptoms that mostly affect the lower parts of the plants and rarely cause plant death. A mild disease form is described in many hop-growing regions^31^, whereas the lethal disease form and the highly virulent *V. nonalfalfae* hop pathotypes have limited distribution in England^32^, Slovenia^33^, and Germany^34^. The longevity of fungal resting structures in soil and the lack of effective chemical control that can cure infected plants are the main obstacles in controlling verticillium wilt^35^. Therefore, disease management relies mainly on phytosanitary measures, soil disinfestation and the use of resistant varieties^36^. At this point in time, it is of utmost importance to study and dissect the pathogenicity and all the related biological processes at many different levels to better understand the pathogen, the host and their interactions to develop new strategies for the defence against verticillium wilt disease. The existence of two pathotypes and of hop varieties with different levels of resistance enables the development of a good model that can be used to obtain a more comprehensive understanding of the pathogenicity of *V. nonalfalfae*.

In the present study, the core components of RNA silencing in *V. nonalfalfae* were investigated, and three RNAi protein groups were identified. Furthermore, expression analysis was performed to examine expression patterns in the highly virulent and less virulent fungal pathotypes and to predict the potential effects of fungal RNAi *in vivo* during *V. nonalfalfae* infection in hop host plants.

## Results

### Identification and structural analysis of the RNAi genes

The homologues of AGO, DCL, and RdRP in the *V. nonalfalfae* genome were identified using BLAST analysis of the available fungal protein sequences for AGO, DCL, and RdRP from the UniProtKB database. All obtained hits were within the predicted gene models^37^, which were subsequently manually curated based on the BLAST results and RNA sequencing (RNA-seq) data^37^. Thus, two AGO, two DCL, and two RdRP genes were identified in the *V. nonalfalfae* genome (Table 1) (GenBank accession numbers MK015618-MK015623). All identified genes were examined using the Conserved Domain Database (CDD), Pfam 31.0, and the Simple Modular Architecture Research Tool (SMART) to predict the conserved domains in AGO, DCL, and RdRP.

**Table 1:** Identified RNAi genes in the *Verticillium nonalfalfae* pathogenic plant fungi.

Protein name	Gene model	Chromosome location	Gene length (bp)	ORF length (bp)	Number of introns	Protein length (aa)	pI	Mw (Da)
VnaAGO1	Vna2.1074	chr 2 4313165–4316218	3054	2943	2	980	9.7	110644.6
VnaAGO2	Vna3.272	chr 3 1089960–1093416	3457	3339	2	1112	9.6	122426.8
VnaDCL1	Vna7.578	chr 7 1938786–1943526	4741	4692	1	1563	6.2	176548.5
VnaDCL2	Vna3.604	chr 3 2396309–2401341	5033	4779	5	1592	6.1	178619.0
VnaRdRP1	Vna5.537	chr 5 2197691–2202374	4684	4626	1	1541	6.4	174679.7
VnaRdRP2	Vna8.563	chr 8 2170393–2174514	4122	4074	1	1357	7.5	152066.4

**Figure 1:**
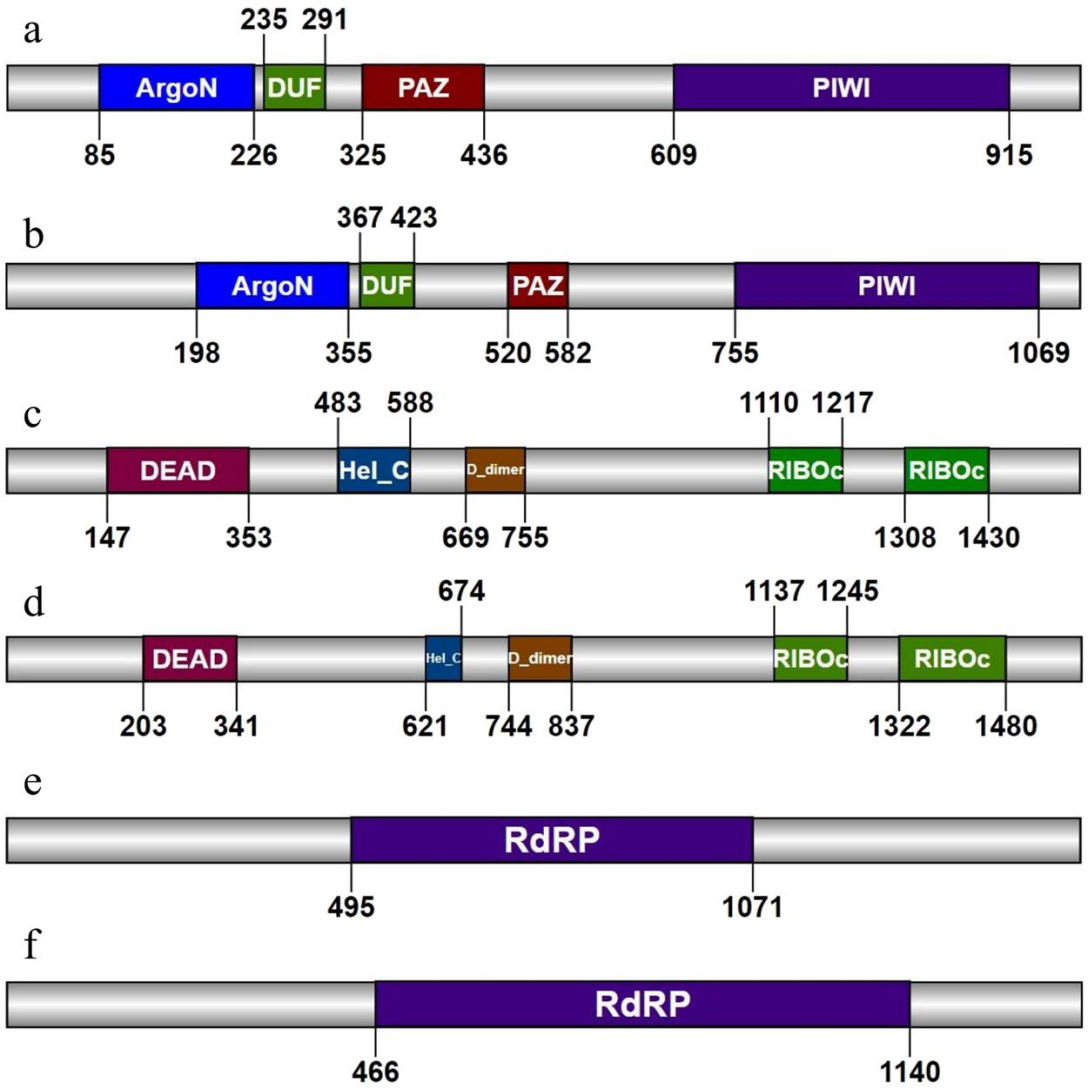
Domain organisation of the six *Verticillium nonalfalfae* RNAi proteins. (**a**) VnaAGO1, (**b**) VnaAGO2, (**c**) VnaDCL1, (**d**) VnaDCL2, (**e**) VnaRdRP1, and **(f**) VnaRdRP2. In the AGO proteins, the following domains were identified: ArgoN – the N-terminal domain of eukaryotic AGOs; DUF – domain of unknown function; PAZ – Piwi-Argonaute-Zwille domain of unknown function in AGO and DCL proteins; PIWI – the double-stranded RNA-guided catalytic domain of AGOs. The DCL proteins contained the following domains: DEAD – the N-terminal DEAD-like helicase; Hel_C – the C-terminal helicase; D_dimer – the Dicer dimerisation domain; RIBOc – the C-terminal ribonuclease III domain. Both RdRP proteins contained the typical RNA-dependent RNA polymerase domain. For all amino acid sequences, the domains were searched for using the Conserved Domain Database, Pfam, and SMART and visualised with IBS (Illustrator of Biological Sequences^55^; http://ibs.biocuckoo.org/online.php).

The *V. nonalfalfae* AGO proteins VnaAGO1 and VnaAGO2, are encoded by the genes Vna2.1074 and Vna3.272, which are located on chromosomes 2 and 3, respectively, and differ in gene and open reading frame length and contain two introns each. The Vna2.1074 gene (3054 bp) encodes the 980-amino acid VnaAGO1 polypeptide, and the Vna3.272 gene (3457 bp) encodes the 1112-amino acid VnaAGO2 polypeptide. The two genes share no significant sequence similarity at the DNA level and share very low identity (23%) at the protein level. Both the VnaAGO1 and VnaAGO2 proteins contain an ArgoN domain (PF16486) at the N-terminus of the protein, which is followed by the DUF (SM01163), PAZ (PF02170), and PIWI (PF02171) domains at the C-terminus (Fig. 1a,b).

Two DCL proteins, VnaDCL1 and VnaDCL2, were identified in the *V. nonalfalfae* genome. Both the Vna7.578 and Vna3.604 genes are of similar length (4741 bp and 5033 bp, respectively) and potentially encode 1563-amino acid (VnaDCL1) and 1592-amino acid polypeptides (VnaDCL2). Both genes differ in the number of introns; the Vna3.604 gene contains five introns, whereas the Vna7.578 gene contains only one intron. At the DNA level, the genes share no significant similarity and only share 22.1% identity at the protein level. Both proteins contain a DExD domain (PF00270) at the N-terminus, which is followed by a helicase-C domain (PF00271), a Dicer-dimerisation domain (PF03368), and two ribonuclease III domains (PF00636) (Fig. 1c,d).

The two identified RdRP genes in *V. nonalfalfae*, Vna5.537 and Vna8.563, differ the most in terms of gene length (4684 bp and 4122 bp, respectively), open reading frame length (4626 bp and 4074 bp, respectively) and the features in the proteins they encode. Vna5.537, which encodes the VnaRdRP1 protein, is translated into an ~170,000 Da polypeptide with a pI of 6.4, whereas the Vna8.563 gene is translated into a ~150,000 Da VnaRdRP2 polypeptide with a pI of 7.6. At the protein level, a 16.9% sequence identity was found, whereas at the DNA level, no significant similarities were found. Both genes contain one short intron, and both RdRP proteins share the conserved RdRP domain (PF05138) (Fig. 1e,f).

**Figure 2:**
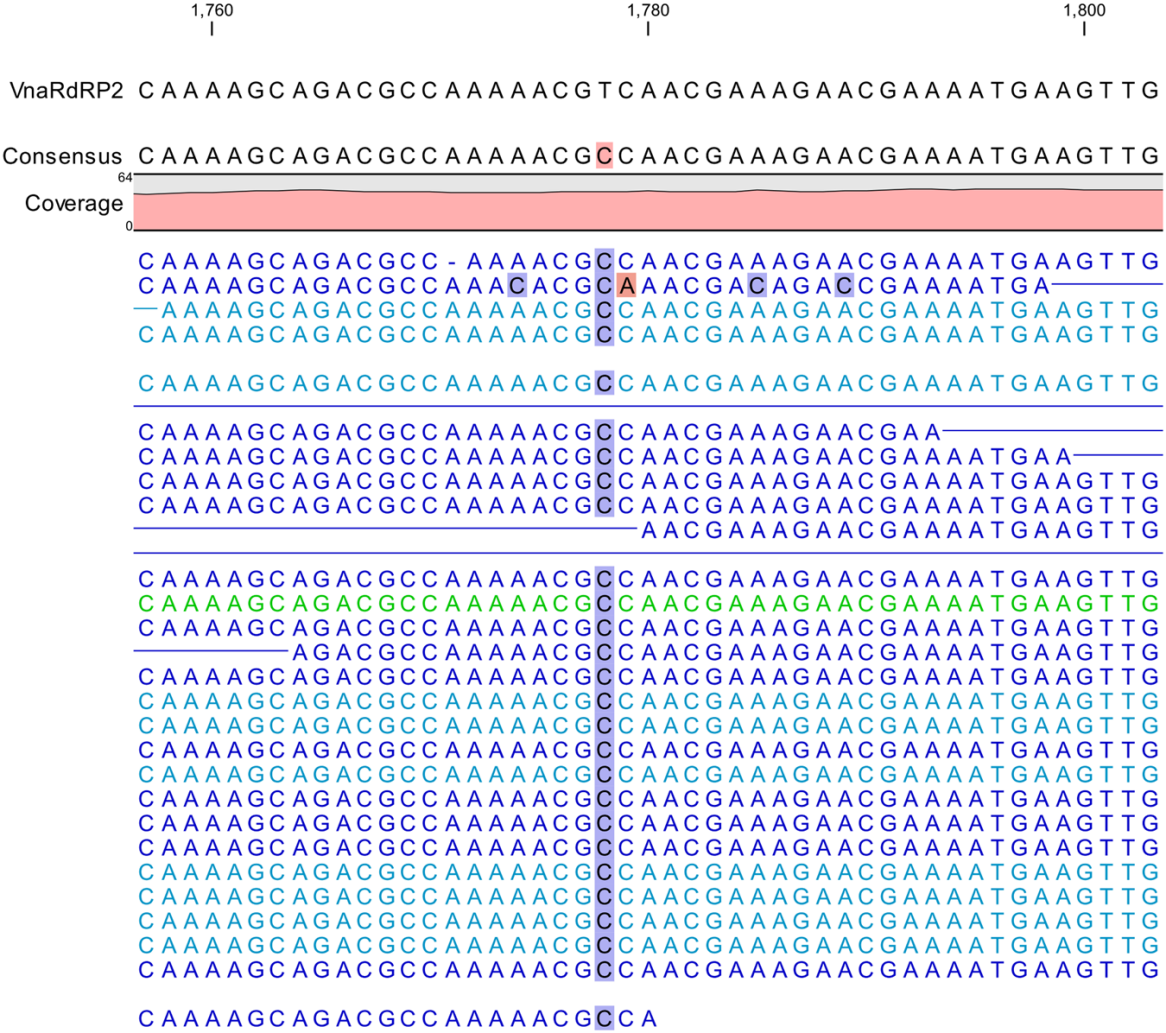
The single nucleotide polymorphism detected in the gene sequence of VnaRdRP2 was present in the less virulent pathotype isolate 1953. The T to C substitution at position 1778, which was detected with the Basic Variant Detection Tool in the CLC Genomic Workbench (ver. 11.0), resulted in a codon change from lysine to proline.

Basic Variant Detection analysis was performed to determine the probable single nucleotide polymorphisms (SNPs) in the *V. nonalfalfae* RNAi genes in the less virulent and highly virulent pathotypes from three geographical origins^36^. Only one SNP (Fig. 2) was identified in the VnaRdRP2 gene of the less virulent *V. nonalfalfae* isolate 1953. A thymine to cytosine substitution was identified at position 1778 in the first exon that results in the conservative replacement of lysine to proline at position 593 in the protein.

### Phylogenetic analysis

Phylogenetic analysis was performed to determine the relationships among the pathogenic plant fungi in the Ascomycete family based on the evolutionarily conserved RNAi pathway. The full-length protein sequences of AGO, DCL, and RdRP for selected pathogenic plant fungi and two Basidiomycete pathogenic plant fungi were retrieved from the NCBI and UniProtKB databases. The protein sequences were aligned using the MUSCLE algorithm, and a maximum likelihood neighbour-joining tree was constructed for the AGO, DCL, and RdRP groups of proteins.

**Figure 3:**
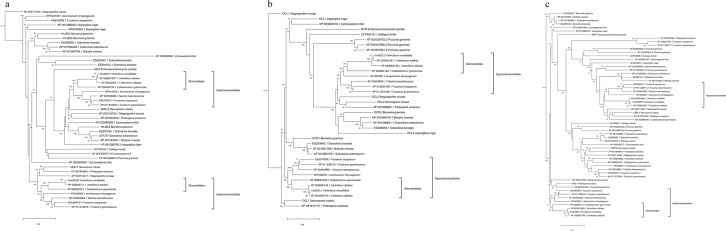
Phylogeny of the Argonaute (AGO), Dicer-like (DCL), and RNA-dependent RNA polymerase (RdRP) proteins for the selected pathogenic plant fungi, including the newly identified *Verticillium nonalfalfae* RNAi proteins. The total protein sequences were aligned using MUSCLE, and maximum likelihood phylogeny based on the WAG protein substitution model was used to construct neighbour-joining trees with 1000 bootstrap replicates for the (**a**) AGO, (**b**) DCL, and (**c**) RdRP groups of proteins. The protein subgroups of fungi belonging to the Glomerellales order, including the newly identified *V. nonalfalfae* AGOs, DCLs, and RdRPs, are marked in each tree.

The AGO neighbour-joining tree contained two distinct groups representing the Ascomycete AGO1 and AGO2 proteins (Fig. 3a). As expected, the two Basidiomycete fungi and *Shizosaccharomyces pombe*, which is a representative fission yeast, formed distinct outgroups and separate clades because they shared less than 34% of sequence similarity with the selected Ascomycete pathogenic plant fungi (Supplementary Fig. S1). In the AGO1 group, VnaAGO1 was grouped together with the XP 003005611.1 *V. alfalfae* and XP 009657937.1 *V. dahliae* proteins and shared 91.9% and 98.6% similarity with them, respectively. The Hypocreomycetidae clade included several proteins from taxonomically related fungi that shared from 44.4% to 58.9% similarity with the VnaAGO1 protein. The AGO2 group comprised proteins similar to the *N. crassa* QDE-2 protein, which was used as a reference. The AGO proteins from *V. nonalfalfae*, *V. dahliae*, *Colletotrichum graminicola* (*C. graminicola*), *Fusarium graminearum* (*F. graminearum*), *Fusarium oxysporum* (*F. oxysporum*), *Nectria haematococcoa* (*N. haematoccoca)*, and *Acremonium chrysogenum* (*A. chrysogenum*) formed the Hypocreomycetidae clade that shared a sequence similarity ranging from 46.3% to 59.8%. The VnaAGO2 protein shared 95.9% sequence similarity with the *V. dahliae* XP 009648711.1 protein to form a *Verticillium* subclade. The phylogenetic tree included several outgroups representing fungi with three or more AGO proteins, including *Zymoseptoria tritici*, *Aspergillius niger*, *Magnaporthe oryzae*, *A. chrysogenum*, and *F. oxysporum*.

Both VnaDCL1 and VnaDCL2 diverged into two clusters that approximately represented the DCL1 and DCL2 fungal proteins (Fig. 3b). In the DCL1 group, VnaDCL1 was grouped together with one *V. dahliae* and one *V. alfalfae* DCL protein (XP 009650143.1 and XP 003009179.1, respectively), since they shared 95.9% and 94.7% sequence similarity, respectively (Supplementary Fig. S2). In the Hypocreomycetidae clade, VnaDCL1 was grouped together with DCL proteins from closely related fungi belonging to the same taxonomic order. The similarity among these proteins ranged from 47.9% to 60.7%. The DCL2 proteins from the Hypocreomycetidae clade shared less sequence similarity, which ranged from 39.8% to 48.7%.

The phylogenetic tree constructed on the basis of the RdRP protein sequences was resolved into five distinct groups of proteins. Two groups included the VnaRdRP1 and VnaRdRP2 proteins. Additional clades represented fungal species with three or more RdRP proteins (Fig. 3c). In the RdRP1 group, the Hypocreomycetidae clade included RdRP proteins from the fungi belonging to the Hypocreomycetidae subclass that had a sequence similarity that ranged from 47.7% to 48.3% (Supplementary Fig. S3). A subclade was formed that included the VnaRdRP1, *V. dahliae* XP 009651476.1, and *V. alfalfae* XP 003007883.1 proteins that shared more than 88.6% sequence similarity. The VnaRdRP2 protein, the *V. dahliae* XP 009655407.1 and *V. alfalfae* XP 003003645.1 proteins formed a typical *Verticillium* subclade. Based on 29.7% to 35.2% sequence similarity, VnaRdRP2 formed a clade comprising proteins from Hypocreomycetidae fungi.

In all the Hypocreomycetidae clades for VnaAGO1/2, VnaDCL1/2, and VnaRdRP1 that represented fungi belonging to the Hypocreomycetidae subclass of Ascomycete fungi, *Colletotrichum graminicola*, which is a representative of the Glomerellaceae family, was grouped together with the *Verticillium* subclade. The amino acid sequence similarity between the investigated *Verticillium* species and *C. graminicola* ranged from 48.7 to 60.7% (Supplementary Figs S1–S3). Based on the protein sequences, the *Verticillium species* and *C. graminicola* formed the Glomerellales clade within the Hypocremoycetidae.

### Expression analysis in different fungal tissues and in *V. nonalfalfae*-infected hop plants

To determine the possible roles of *V. nonalfalfae* RNAi proteins in the development and pathogenicity of the fungi, expression analysis was performed in conidia and two different mycelia sources for both fungal pathotypes using quantitative RT-PCR (qRT-PCR). Additionally, the expression analysis of RNAi genes in the *V. nonalfalfae*-infected hop plants was performed to elucidate the potential importance of the RNAi to pathogenicity during the infection of plants.

**Figure 4:**
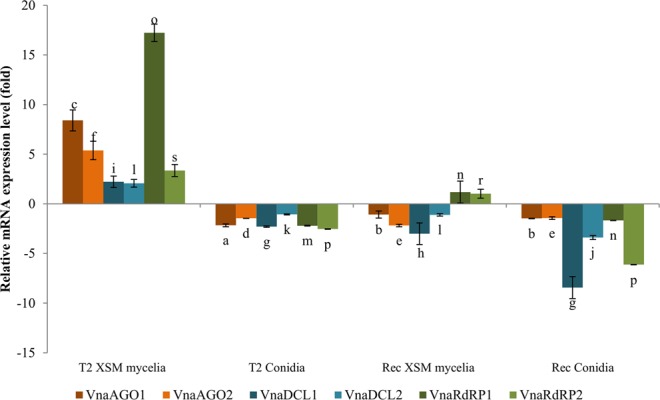
Expression levels of the *V. nonalfalfae* RNAi genes in the conidia and XSM mycelia of the highly virulent T2 and less virulent Rec pathotypes; the results are shown as fold-changes relative to the level of expression in the mycelia grown in CD. The statistical significance of the difference in the expression of each gene investigated in conidia and XSM mycelia in the less virulent Rec and highly virulent T2 isolates is denoted with letters (**a**–**c**) for VnaAGO1, (**d**–**f**) for VnaAGO2, (**g**–**i**) for VnaDCL1, (**j**–**l**) for VnaDCL2, m-o for VnaRdRP1, and p-s for VnaRdRP2; the same letters indicate no statistical difference at the significance level of *P* < 0.05 in the expression of an investigated gene in the two pathotypes and in the two investigated fungal tissues.

All *V. nonalfalfae* RNAi genes were expressed in both of the investigated fungal tissues, conidia and mycelia grown in xylem-simulating media (XSM) (Fig. 4); however, the expression levels varied between the less virulent isolate Rec and the highly virulent isolate T2. The highest expression levels of the AGO, DCL, and RdRP genes were encountered in the XSM mycelia of the highly virulent pathotype T2, where all investigated genes exhibited higher expression levels in comparison with their expression in the mycelia grown in Czapek Dox (CD) media. In the case of VnaRdRP1, its gene expression was upregulated 17-fold, and for VnaAGO1/2, VnaDCL1/2, and VnaRdR2 the gene expression levels were upregulated 2- to 8.4-fold (Supplementary Table S1). In the mycelia grown in XSM for the less virulent isolate Rec, only VnaRdRP1/2 were slightly upregulated, and the expression of all the VnaAGO1/2 and VnaDCL1/2 genes was lower compared to those in mycelia grown in CD for the Rec isolate. In conidia of the T2 and Rec isolates, the expression levels of all genes were lower than those in mycelia grown in CD for both the highly virulent isolate T2 and the less virulent isolate Rec.

**Figure 5:**
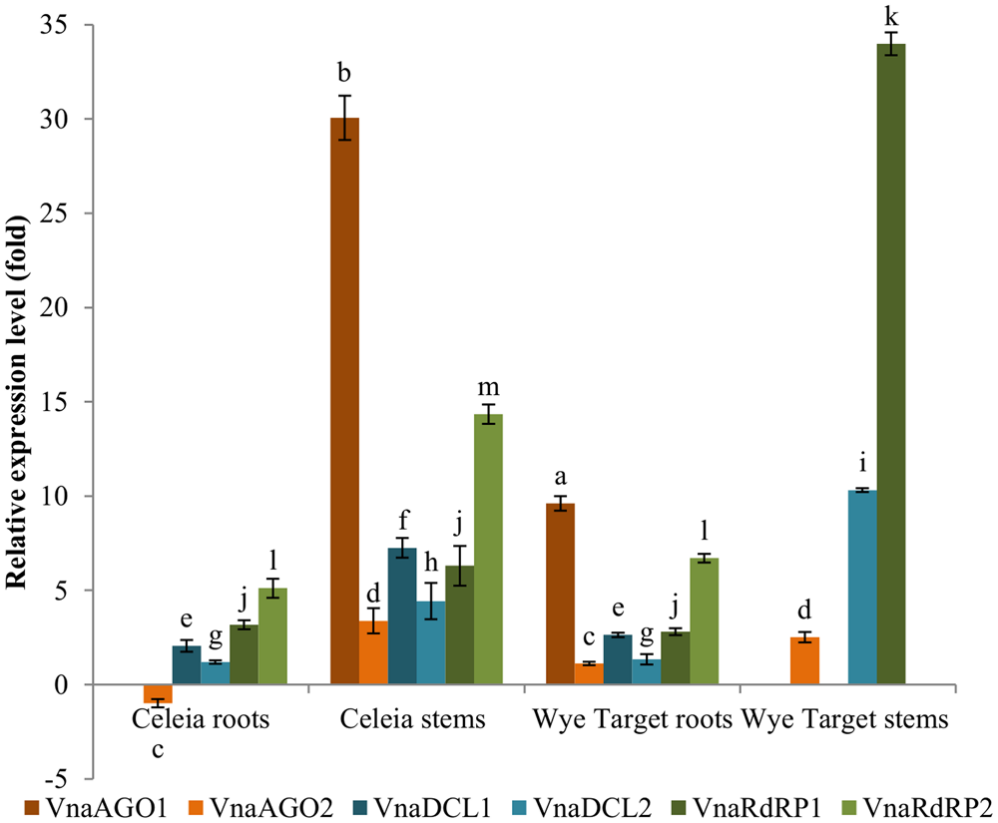
Expression levels of the *V. nonalfalfae* RNAi genes in the T2-infected hop roots and stems of the susceptible Celeia and resistant Wye Target cultivars; the results are shown as fold-changes relative to the expression in the mycelia grown in CD from the isolate T2. The statistical significance of the difference in the expression of each gene in the roots and stems of the *V. nonalfalfae*-infected Celeia and Wye Target cultivars is denoted with letters (**a**,**b**) for VnaAGO1, (**c**,**d**) for VnaAGO2, (**e**,**f**) for VnaDCL1, (**g**–**i**) for VnaDCL2, (**j**,**k**) for VnaRdRP1, and (**l**,**m**) for VnaRdRp2; the same letters indicate no statistical difference at the significance level of *P* < 0.05 in the expression of an investigated gene in the two cultivars or in the two investigated hop tissues.

In hop plants infected with the highly virulent *V. nonalfalfae* isolate T2, different expression levels were observed for the fungal RNAi genes in the roots and stems of the *V. nonalfalfae*-susceptible Celeia and *V. nonalfalfae*-resistant Wye Target cultivars (Fig. 5). The relative expression levels of the RNAi genes were calculated in comparison with their expression in mycelia grown in CD for the highly pathogenic isolate T2. In the stems of the susceptible Celeia cultivar, all fungal RNAi genes were abundantly expressed, with fold increases ranging from 3.4 to 30.1 (Supplementary Table S2). However, in the resistant hop cultivar, the highest expression levels were observed in the stems for VnaDCL2 and VnaRdRP1 (upregulated 10.3- and 34-fold, respectively). Interestingly, in the stems of the resistant hop cultivar, VnaAGO1 was not expressed, and VnaAGO2 showed only 2.5-fold upregulation. In the roots of both the resistant Wye Target and the susceptible Celeia cultivars, all studied RNAi genes were differentially expressed, with the highest expression levels observed for VnaDCL1 and VnaRdRP1/2. In the case of the AGO genes, only VnaAGO1 was significantly upregulated by 9.6-fold in the resistant cultivar, whereas in the susceptible cultivar, VnaAGO1 was not expressed, and VnaAGO2 was slightly downregulated.

## Discussion

The RNAi mechanism is evolutionarily conserved in the fungal kingdom and is mediated by the actions of the AGO, DCL, and RdRP proteins; only a few species, such as the model organism *Saccharomyces cerevisiae* and the highly virulent human pathogen *Cryptococcus deuterogattii*, have been reported to have lost this system^16,38^. On average, fungal species belonging to the Pezizomycotina subphylum, which comprises filamentous ascomycete fungi, contain two AGO, two DCL, and three RdRP genes in their genome^38^, and their RNAi mechanisms have been implicated in growth, development, conidiation, responses to biotic and abiotic stress and pathogenicity^23,39,40^. In the case of *V. nonalfalfae*, all three groups of RNAi core genes were identified: two AGOs, two DCLs, and two RdRPs.

All six *V. nonalfalfae* RNAi proteins share common features with other fungal RNAi proteins and contain all the necessary conserved functional domains (Fig. 1). Both *V. nonalfalfae* DCL proteins had conserved domains typical of the class III endonuclease family^16^; however, both proteins lacked the PAZ domain, similarly to the *N. crassa* reference RNAi proteins DCL-1 and DCL-2^15^. The same phenomenon has been observed in other fungal Dicer proteins identified in *Trichoderma atroviride*^39^, *F. graminearum*^23^, *Metarhizium robertsii*^24^, and *M. oryzae*^40^. On the other hand, both the VnaDCL1 and VnaDCL2 proteins contained a divergent double-stranded RNA binding domain, which is newly referred to as the Dicer-dimerisation domain but was formerly known as the DUF283 domain (dicer domain of unknown function)^41^.

The AGO proteins in *V. nonalfalfae* contain ArgoN, DUF, PAZ and PIWI domains, which are conserved in the Argonaute family. The MID and PIWI domains are referred to as the PIWI-superfamily domain (cl00628), which contain the 5′ anchoring subdomain for the guide RNA and the catalytic subdomain at the C-terminus of the protein^42^. The same domain construction is observed in the *N. crassa* QDE-2 protein that was used as a reference for the fungal AGO proteins. Both *V. nonalfalfae* RdRP proteins contain the typical RdRP domain that is present in the eukaryotic RNA-dependent RNA polymerases, which was also found in the *N. crassa* QDE-1 reference protein.

Phylogenetic analysis performed in the selected group of pathogenic plant fungi revealed a high level of conservation of the RNAi system in the Sordariomycetes taxonomic class of Ascomycete fungi (Fig. 3). As revealed by the amino acid sequence pairwise comparison that was performed for each group of RNAi proteins, the proteins shared approximately 40% amino acid sequence similarity in the case of VnaAGO1/2 and VnaDCL1/2. The Dicer neighbour-joining tree clearly illustrated the relatedness of the selected fungal species at both physiological and taxonomical levels, as the members of the Hypocreomycetidae subclass, including *A. chrysogenum*, *C. graminicola*, *N. haematococca*, *F. graminearum*, *F. oxysporum* and the three *Verticillium* species, formed distinct clades according to a confirmed taxonomy that was supported by high bootstrap values (Fig. 3). The members of the Hypocreomycetidae taxa live primarily as saprotrophs, but the selected fungi all represented severe and highly virulent soil-borne pathogens of plants that typically exploit host nutrients through intra-host growth. The new taxonomy of the *Verticillium* species placed the *Verticillium* sensu stricto genus in the Plectosphaerellaceae family that, together with the Glomerellaceae family, comprises the Glomerellales taxonomic order^29^. The close relatedness of both Glomerellales families is illustrated in all three phylogenetic trees, where the three *Verticillium* species and the representative of Glomerellaceae, *C. graminicola*, formed the Glomerellales clade within the Hypocreomycetidae subfamily.

Interestingly, in the case of *V. dahliae*, which is another representative of the *Verticillium* sensu stricto genus, three RdRP proteins were identified (Bioproject PRJNA28529), whereas in *V. nonalfalfae*, a homolog of the third RdRP protein (XP 009654829.1) from the morphologically distinct *V. dahliae* species, which is characterised by typical microsclerotia resting structures^29^, was not found in the *V. nonalfalfae* genome using our identification and characterisation approach. Moreover, both *V. alfalfae* and *V. nonalfalfae* contained only two RdRP proteins. No morphological differences existed between *V. alfalfae* and *V. nonalfalfae*. Both species were formerly named *V. albo-atrum*; however, recent molecular marker analysis revealed differences at the DNA level, and thus *V. albo-atrum* was divided into three species: *V. alfalfae*, *V. nonalfalfae*, and the true *V. albo-*atrum^29^.

A similar number of RNAi genes were identified in *F. graminearum*, which is a close relative of *V. nonalfalfae* that has been extensively studied because of its devastating impact on wheat and barley production. The sequence similarity was as high as 50% for the AGO and DCL proteins; however, only two RdRP proteins were identified in the *V. nonalfalfae* genome, whereas the *F*. *graminearum* genome contains five RdRP proteins^23^. Interestingly, only two *F. graminearum* RdRP proteins appeared to be closely related to the *N. crassa* QDE-1 protein, and one of these RdRP proteins has been reported to be related to the plant RdRP-like protein, indicating that the genes evolved from different ancestors or perhaps that even horizontal transfers occurred^23^. One *F. graminearum* RdRP, FgRdRP3, is homologous to RRP3, which is the third RNA-dependent RNA polymerase identified in the *N. crassa* genome^43^. In *N. crassa*, a second RNA-dependent RNA polymerase, SAD-1, exists and has been implicated in the meiotic silencing pathway by unpaired DNA (MSUD) mechanism, which is a second silencing mechanism in *N*. crassa^43^. Phylogenetic analysis performed in the RdRP group of proteins revealed the existence of a SAD-1-like protein in *V. nonalfalfae*, as the RdRP domain sequence in VnaRdRP1 shared 56.7% similarity with the *N. crassa* SAD-1 RdRP domain. The MSUD mechanism protects the genome from all DNA sequences that do not pair during prophase I of meiosis in sexual reproduction^44^. MSUD-associated siRNAs derived from transposons have been reported, implicating MSUD in genome defence during meiosis^45^. Pairwise comparison of all the fungal AGO genes included in the phylogenetic analysis also revealed 37.3% amino acid sequence similarity between VnaAGO1 and SMS-2 (meiotic silencing suppressor 2), which is a second protein in *N. crassa* that belongs to the AGO family^43^ and represents the AGO protein in the MSUD pathway. The amino acid similarity between VnaRdRP1/SAD-1 and VnaAGO1/SMS-2 indicated that *V. nonalfalfae* may use the MSUD silencing pathway to protect the genome and ensure its stability; however, fungi belonging to the *Verticillium* sensu stricto genus reproduce asexually^29^, so this mechanism is unlikely to be present in *V. nonalfalfae*. Nevertheless, recent studies in different lineages of *V. dahliae* have demonstrated that in *V. dahliae*, both fungal mating types exist, as well as some meiosis-specific genes^46^. Furthermore, the different lineages appear to have evolved via the recombination of sexual ancestors, suggesting that these fungi may still possess the mechanisms needed to reproduce sexually or may have only recently lost this ability^46^. With that in mind, the existence of the MSUD pathway in *V. nonalfalfae* is more plausible; however, additional studies must be performed to elucidate the roles of VnaAGO1 and VnaRdRP1 in fungal development.

Recent studies have revealed that fungal pathogens use RNAi not only as a genome integrity tool and a mechanism for adapting to environmental stresses^25^ but also as one of the virulence factors that contribute significantly to host colonisation by suppressing host immune responses^9,11,25,47^. Thus, the expression analysis of RNAi genes in a culture of *V. nonalfalfae* under simulated xylem conditions and *in-planta* during the infection of hops was performed. The results support the existence of fungal RNAi activity during the infection process; however, the trans-kingdom RNAi between the host and the pathogen must be experimentally deciphered.

The highest expression levels of all six *V. nonalfalfae* RNAi genes were observed in the highly virulent pathotype isolate T2, in which all the genes were upregulated in the mycelia grown in XSM media (Fig. 4), which simulated the conditions in plant xylem tissue in which fungi actively spread in the host^48^. On the other hand, in the mild pathotype Rec, these genes were not significantly upregulated or downregulated in the mycelia grown in XSM media, suggesting the role of RNA silencing in the invasion of the plant by the highly virulent pathotype. It has been discovered that *Botrytis cinerea* exports siRNAs to host plants, where they act as effectors to disrupt plant immune responses^10^. However, to the best of our knowledge, the extent of the silencing processes in *V. nonalfalfae* and whether the fungi actively silence endogenous genes to compromise growth to ensure enhanced pathogenicity or if they use the silencing mechanism as a pathogenicity tool that involves the synthesis and export of small RNAs into the host, remain unknown.

Different studies of plant-pathogen interactions have proposed that the plant vasculature is the target site for the study of RNAi signalling between plants and fungal pathogens that is mediated by extracellular vesicular transport^11,49^. Thus, the focus of our study shifted from fungi to *V. nonalfalfae*-infected hop plant hosts. Two tissues were examined for the expression of fungal RNAi genes: the roots, where the host and the pathogenic fungi first interact, and the stems, where the clogging of the vascular tissue that causes wilting takes place^29^. Interestingly, in the stems of both resistant Wye Target and susceptible Celeia hop cultivars, the fungal RNAi genes were upregulated to a higher degree compared to the expression in the roots. However, two distinct differences were observed, leading us to believe that the fungi could use RNAi in a different manner during the infection of susceptible or resistant hops. In the latter scenario, VnaRdRP1 was upregulated by 34-fold and VnaDCL2 by 10.3-fold in the stems, indicating that the fungi actively synthesise siRNAs during infection. On the other hand, only one AGO gene, VnaAGO2, was upregulated to a lower degree, suggesting that the fungi produce siRNAs in the stems of resistant hops; however, since the AGOs are not strongly expressed (VnaAGO1 is not expressed at all), the siRNAs could be exported to hops as effectors to mediate the host immunity responses. In the stems of the susceptible cultivar, VnaAGO1 was upregulated by 30-fold and VnaAGO2 by 3.4-fold, leading us to conclude that in the stems of the susceptible cultivar, the fungi likely use RNAi to mediate the expression of endogenous genes.

The data in the present study suggest a plausible role of RNAi in the pathogenesis of *V. nonalfalfae* fungi, as demonstrated by high expression levels of all fungal RNAi genes in the highly virulent pathotype and in infected hops. The mechanism involved and its components are highly conserved among the most extensively studied Sordaryomycete pathogenic plant fungi. In the future, studies of fungal small RNAs and their possible export to hop vascular tissue will be performed. Moreover, the construction of deletion mutants for AGOs, DCLs, and RdRPs could provide better knowledge of the involvement of RNAi in the pathogenesis of *V. nonalfalfae* and the host-pathogen interactions.

## Methods

### Available data

The reference genome of the highly virulent *V. nonalfalfae* isolate T2 and the computationally annotated gene models are available from our laboratory^37^ (BioProject PRJNA283258, BioSample SAMN03610599), including the whole-genome shotgun (WGS) reads for the other five isolates from three distinct geographical origins: two addition highly virulent isolates, P55 (Germany, SRR2013835) and 1985 (England, SRR2013863), and three less virulent isolates, Rec (Slovenia, SRR2012749 and SRR2012748), P15 (Germany, SRR2013806), and 1953 (England, SRR2013836); in addition, RNA-seq data from three biological replicates of the T2 (SRX1020629) and Rec (SRX1020679) isolates is available. All data and the WGS master data are available in the SRA database.

### Identification of the core RNAi genes

For the selected pathogenic plant fungi (Table 2), including *N. crassa* as a fungal RNAi model, the amino acid sequences of the AGO, DCL, and RdRP proteins were downloaded from the UniProtKB database (http://www.UniProt.org/) and used as reference sequences for a TBLASTN comparison against the annotated *V. nonalfalfae* genome^37^. The hits with the highest e-values and >95% protein coverage were selected for further analysis. *V. nonalfalfae* RNA-seq data were mapped onto *V. nonalfalfae* genomic sequences^37^ to support the manual gene model curation. The fungal gene sequences were submitted to GenBank. The protein sequence prediction was performed using NCBI ORFfinder (https://www.ncbi.nlm.nih.gov/orffinder/). The complete amino acid sequences were further characterised to identify their functional domains using CDD (https://www.ncbi.nlm.nih.gov/Structure/cdd/wrpsb.cgi), Pfam 31.0 (https://pfam.xfam.org), and SMART (http://smart.embl.de). For AGO protein candidates, the ArgoN (PF16486), PAZ (PF02170), and PIWI (PF02171) domains were searched. The DExD (PF00270), Helicase_C (PF00271), and RIBOc III (PF00636) domains were searched for within the DCL protein candidates. For the RdRP candidates, the RNA-dependent RNA polymerase domain (PF05183) was searched for within the amino acid sequence.

**Table 2:** Pathogenic plant fungi included in the phylogeny.

Species name	Phylum	Class	Subclass	Order	Family	No. of AGOs	No. of DCLs	No. of RdRPs
*Puccinia graminis*	Basidiomycota	Pucciniomycetes		Pucciniales	Pucciniaceae	2	3	4
*Ustilago hordei*	Basidiomycota	Ustilaginomycetes	Ustilaginomycetidae	Ustilaginales	Ustilaginaceae	1	1	2
*Schizosaccharomyces pombe*	Ascomycota	Schizosaccharomycetes		Schizosaccharomycetales	Schizosaccharomycetaceae	1	1	1
*Aspergillus niger*	Ascomycota	Eurotiomycetes	Eurotiomycetidae	Eurotiales	Aspergillaceae	3	2	2
*Zymoseptoria tritici*	Ascomycota	Dothideomycetes	Dothideomycetidae	Capnodiales	Mycosphaerellaceae	3	1	3
*Blumeria graminis*	Ascomycota	Leotiomycetes	Leotiomycetidae	Erysiphales	Erysiphaceae	3	2	1
*Sclerotinia borealis*	Ascomycota	Leotiomycetes	Leotiomycetidae	Helotiales	Sclerotiniaceae	4	2	3
*Sclerotinia sclerotiorum*	Ascomycota	Leotiomycetes	Leotiomycetidae	Helotiales	Sclerotiniaceae	2	2	3
*Botrytis cinerea*	Ascomycota	Leotiomycetes	Leotiomycetidae	Helotiales	Sclerotiniaceae	2	2	3
*Colletotrichum graminicola*	Ascomycota	Sordariomycetes	Hypocreomycetidae	Glomerellales	Glomerellaceae	2	2	3
*Magnaporthe oryzae*	Ascomycota	Sordariomycetes	Sordariomycetidae	Magnaporthales	Magnaporthaceae	3	2	3
*Podospora anserina*	Ascomycota	Sordariomycetes	Sordariomycetidae	Sordariales	Lasiosphaeriaceae	2	2	4
*Neurospora crassa*	Ascomycota	Sordariomycetes	Sordariomycetidae	Sordariales	Sordariaceae	2	2	3
*Fusarium graminearum*	Ascomycota	Sordariomycetes	Hypocreomycetidae	Hypocreales	Nectriaceae	2	2	5
*Fusarium oxysporum*	Ascomycota	Sordariomycetes	Hypocreomycetidae	Hypocreales	Nectriaceae	3	2	4
*Nectria haematococca*	Ascomycota	Sordariomycetes	Hypocreomycetidae	Hypocreales	Nectriaceae	2	2	4
*Acremonium chrysogenum*	Ascomycota	Sordariomycetes	Hypocreomycetidae	Hypocreales	Acremonium	3	2	3
*Verticillium dahliae*	Ascomycota	Sordariomycetes	Hypocreomycetidae	Glomerellales	Plectosphaerellaceae	2	2	3
*Verticillium alfalfae*	Ascomycota	Sordariomycetes	Hypocreomycetidae	Glomerellales	Plectosphaerellaceae	1	2	2
*Verticillium nonalfalfae*	Ascomycota	Sordariomycetes	Hypocreomycetidae	Glomerellales	Plectosphaerellaceae	2	2	2

### Polymorphism analysis

The identified *V. nonalfalfae* RNAi gene sequences were used for the Basic Variant Detection analysis in the CLC Genomic Workbench (ver. 11.0.1) using the default settings. Single nucleotide polymorphisms in VnaAGO1/2, VnaDCL1/2, and VnaRdRP1/2 were searched for in WGS data from six *V. nonalfalfae* isolates that represented both less virulent (Rec, 1953 and P55) and highly virulent pathotypes (T2, 1985 and P15) of fungi of different geographical origins in Slovenia, England and Germany, respectively.

### Phylogenetic analysis

The total protein sequences of AGOs, DCLs, and RdRPs from selected pathogenic plant fungi (Table 2), including the sequences of identified *V. nonalfalfae* RNAi proteins, were used for the phylogenetic analysis. Multiple sequence alignments were performed using the MUSCLE algorithm^50^ implemented in the CLC Genomics Workbench (ver. 11.0.1) for each of the three groups of protein sequences. The alignments were manually edited to trim all positions containing gaps and missing data. The maximum likelihood method based on the WAG protein substitution model^51^ and implemented in the CLC Genomics Workbench was used for the phylogenetic tree construction (neighbour-joining (NJ) tree construction method), and the reliability of the tree nodes was tested with 1000 bootstrap replicates. The phylogenetic trees were visualised using MEGA software (ver. 10.0.5). The pairwise analysis of the aligned amino acid sequences for each group of proteins was performed in the CLC Genomics Workbench to determine the sequence identity percentages.

### Fungal and hop materials and growth conditions

The two isolates of *V. nonalfalfae* that represented less virulent (isolate Rec) and highly virulent (isolate T2)^35^ pathotypes that were used in the present study were obtained from the culture collection of the Slovenian Institute for Hop Research and Brewing. For mycelial production, cultures were grown in an incubator for 6 days at 25 °C and 120 rpm in liquid Czapek Dox (CD) broth (Sigma-Aldrich) and in XSM prepared according to the method described by Neumann and Dobinson^48^. For conidia production, cultures were grown in Petri dishes containing CD broth solidified with 0.8% agar (Duchefa) and kept at room temperature in the dark. The conidia were harvested following two weeks of growth, and all tissue samples were frozen in liquid nitrogen and stored at −80 °C.

*In-planta* expression analysis was performed using hop plants infected with the highly virulent *V. nonalfalfae* isolate T2. The hop plants, fungal inoculum, and inoculation and growth conditions used were as described in the protocol by Flajšman *et al*.^52^. In brief, the roots of two-month-old, well-rooted Verticillium wilt-susceptible Celeia and resistant Wye Target cultivars were dipped in fungal inoculum containing 5 × 10^6^ conidia/mL and grown in a chamber under controlled light (12-h photoperiod of fluorescent light) at a temperature of 20–25 °C and in 65–70% humidity. The roots and stems were harvested at 1, 3, 6, 12, 15, 18, and 30 days post-inoculation (dpi), and all material was ground in liquid nitrogen and stored at −80 °C.

### RNA extraction and RT-PCR analysis

For each fungal pathotype, RNA was extracted from conidia and mycelia grown in CD broth and mycelia grown in XSM from three biological replicates. For the infected hops, RNA was extracted from roots harvested at 1 dpi for both susceptible and resistant cultivars and from stems harvested at 30 dpi for the susceptible cultivar and at 15 dpi for the resistant cultivar for three biological replicates, which is when the relative amount of fungi in the hop stems was at the highest point (data unpublished). RNA was isolated using a Spectrum™ Plant Total RNA Kit (Sigma-Aldrich) according to the manufacturer’s protocol, and the genomic DNA was removed using an On-Column DNase I Digestion Set (Sigma-Aldrich). The quality and quantity of RNA were assessed using an Agilent 2100 Bioanalyzer and an RNA 6000 Nano Kit (Agilent Technologies) to determine the concentration and integrity (RIN) of the isolated total RNA. First strand cDNA was synthesised using a High Capacity cDNA Reverse Transcription Kit (Applied Biosystems) with RNA extracted from the fungal material and with a GoScript™ Reverse Transcription System (Promega) for the isolated hop RNA according to the manufacturers’ instructions. Specific primers for each target gene were designed using Primer Express 3.0 software or the Primer3 online tool (http://bioinfo.ut.ee/primer3-0.4.0/) (Supplementary Table S3) and analysed using OligoAnalyzer (https://eu.idtdna.com/calc/analyzer). Previously developed reference gene sequences for *V. nonalfalfae*^53^, Vna8.801 (splicing factor 3a2) and VnaUn.148 (DNA topoisomerase) were used as internal controls for data normalisation. Quantitative RT-PCR was performed using the 7500 Real-Time PCR System and software version 2.3 (Applied Biosystems). For each biological sample, qPCR was performed for three replicates. The relative expression levels of the target genes were calculated based on the mathematical model proposed by Pfaffl^54^. Briefly, the average delta cycle threshold value (dCt) for the less virulent Rec and highly virulent T2 isolates, which was normalised to the expression of the reference genes, was used for the calculation of ddCt. The expression levels in CD mycelia were selected as an internal control for the calculation of the fold-change in the conidia and XSM mycelia. For *in-planta* expression analysis, raw Ct values for each gene expressed in the roots and stems of the most abundantly colonised plants were normalised to the average Ct value of the reference gene, and the colonisation factor was applied to normalise the fungal RNA content. The ddCt was calculated using the dCt value of the CD mycelia from the highly virulent isolate T2 as an internal control, and the expression levels are shown as logarithmic fold-change values. The statistical significance of the expression patterns was analysed using one-way analysis of variance (one-way ANOVA) at a significance level of *P* < 0.05.

## Supplementary information

RNA interference core components identified and characterised in Verticillium nonalfalfae, a vascular wilt pathogenic plant fungi of hops

## Data Availability

All data generated or analysed during this study are included in this published article (and the Supplementary Information files). The datasets generated during the current study were submitted to the GenBank repository under accession numbers MK015618-MK015623.
