# Supplementary material for: RNA interference core components identified and characterised in Verticillium nonalfalfae, a vascular wilt pathogenic plant fungi of hops
Source: Sci Rep. 2019 Jun 17;9:8651. doi: 10.1038/s41598-019-44494-8 (PMC6572790; doi:10.1038/s41598-019-44494-8)
Supplement: Supplementary file 1 — RNA interference core components identified and characterised in Verticillium nonalfalfae, a vascular wilt pathogenic plant fungi of hops [file 41598_2019_44494_MOESM1_ESM.docx]

**Supplementary documents**

**RNA interference core components identified and characterised in *Verticillium nonalfalfae*, a vascular wilt pathogenic plant fungi of hops**

Taja Jeseničnik^1^, Nataša Štajner^1^, Sebastjan Radišek^2^, and Jernej Jakše^1,*^

Agronomy Department^1^, Biotechnical Faculty, University of Ljubljana, Ljubljana, Slovenia; Slovenian Institute of Hop Research and Brewing^2^, Žalec, Slovenia

Corresponding author: Jernej Jakše, PhD

Professor, Agronomy Department, Biotechnical Faculty, University of Ljubljana, Slovenia

Jamnikarjeva 101, Ljubljana, 1000, Slovenia

Phone: 00386 1 3203 280

Fax: 4231088

E-mail: Jernej.Jakse@bf.uni-lj.si

Short running head: RNA interference core components in *Verticillium nonalfalfae*

**Supplementary Figure 1. Pairwise comparison of the amino acid sequences of the AGO proteins for selected pathogenic plant fungi; sequence similarities are indicated as percentage amino acid identities.**

**
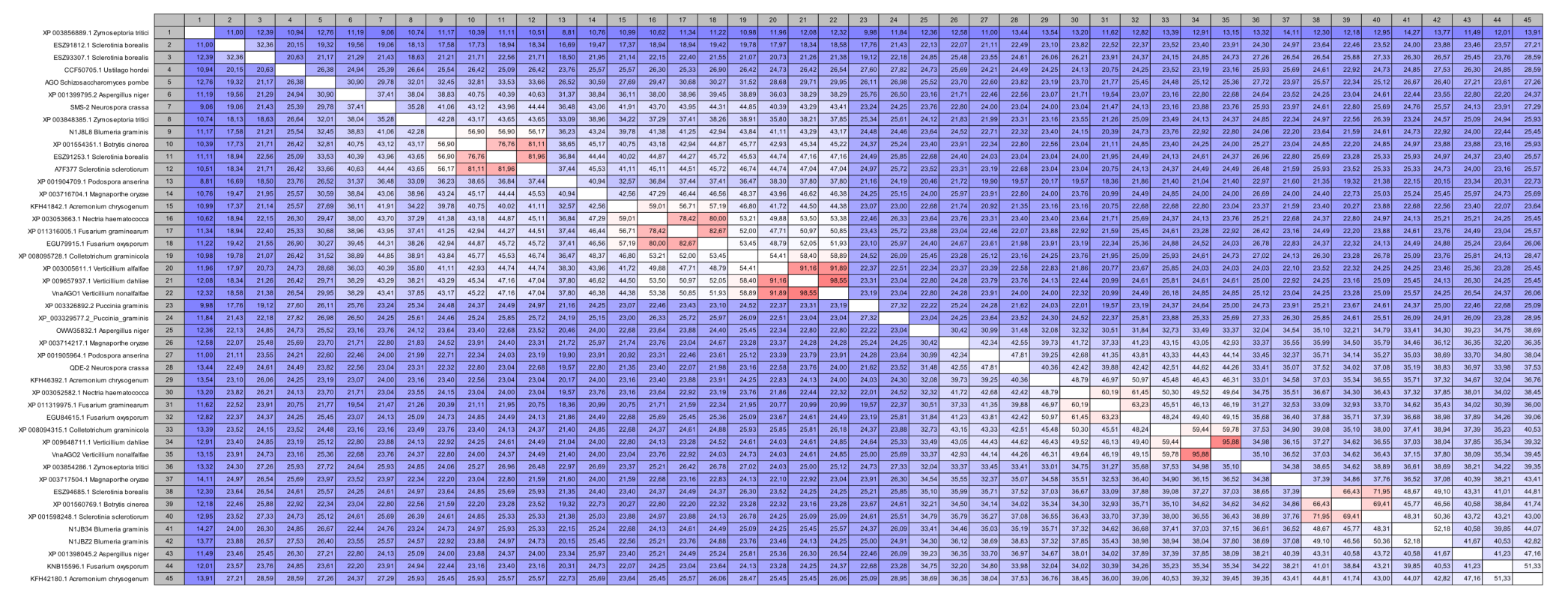
**

**Supplementary Figure 2. Pairwise comparison of the amino acid sequences of the DCL proteins for selected pathogenic plant fungi; sequence similarities are indicated as percentage amino acid identities.**


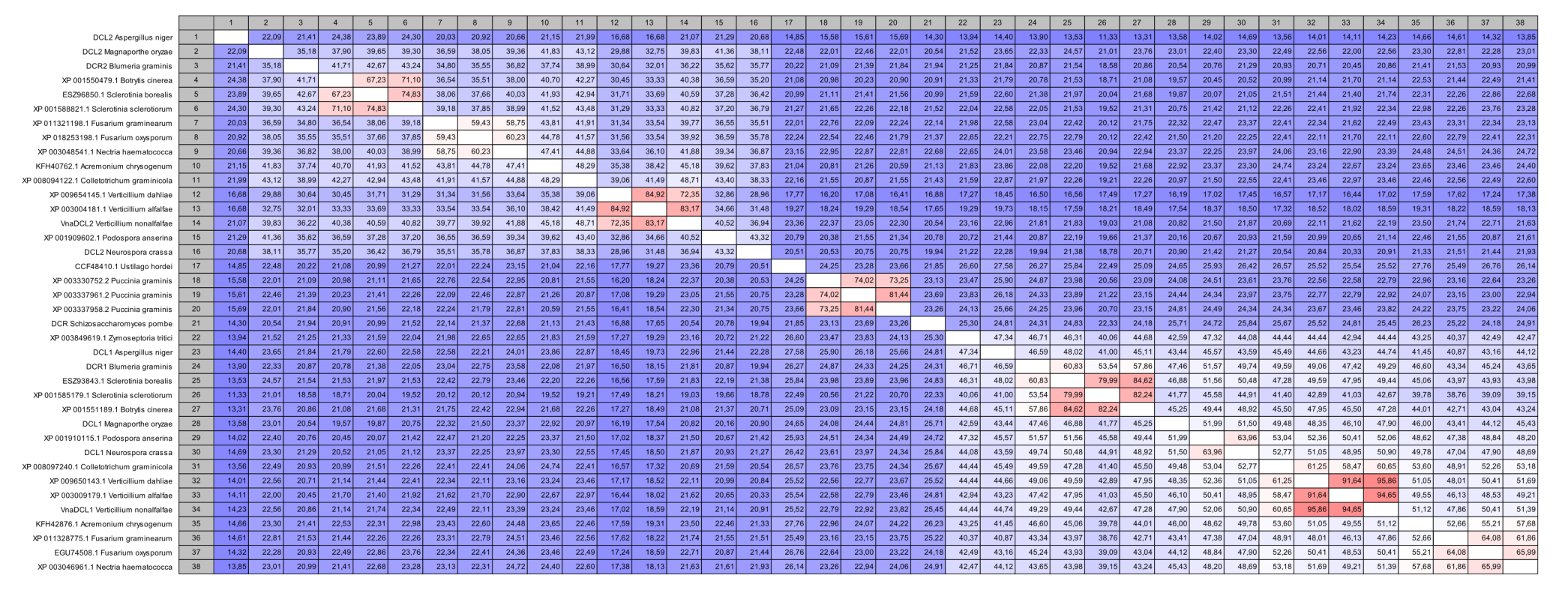


**Supplementary Figure 3. Pairwise comparison of the amino acid sequences of the RdRP proteins for selected pathogenic plant fungi; sequence similarities are indicated as percentage amino acid identities.**

**
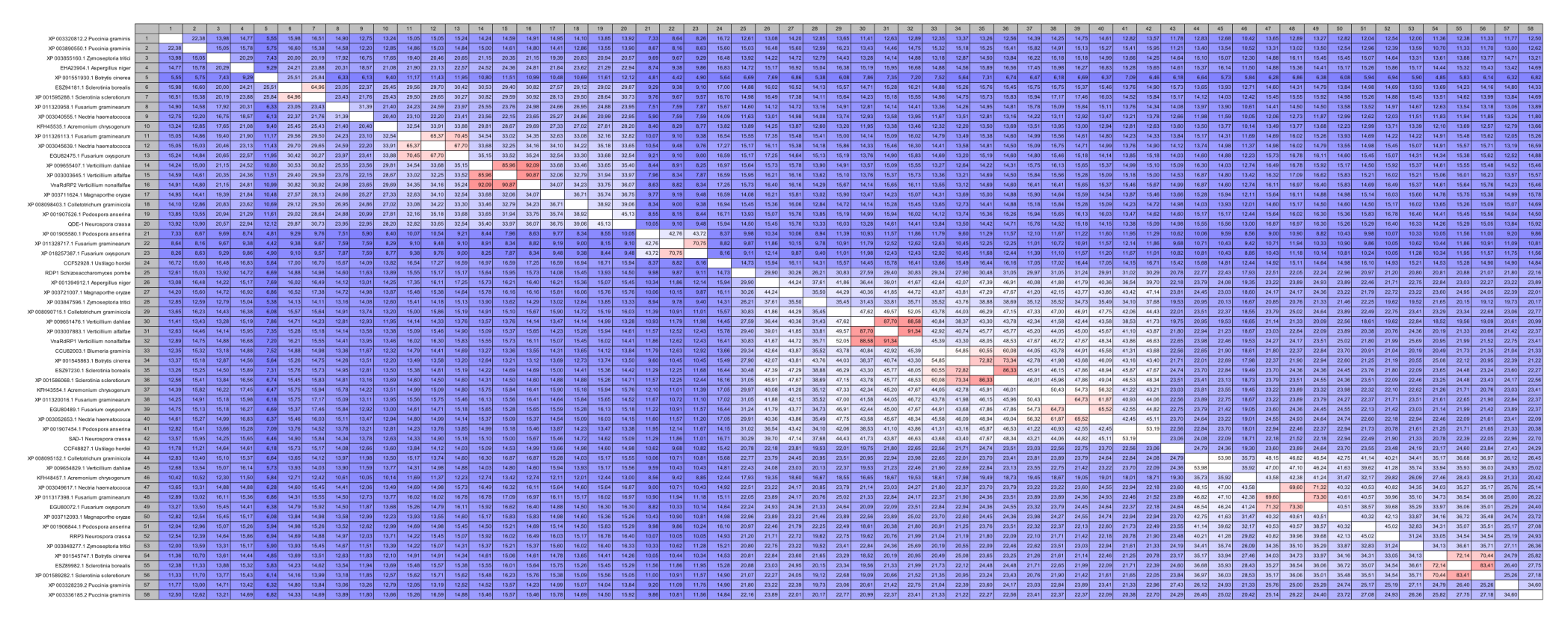
**

Supplementary Table 1. Expression levels of the *V. nonalfalfae* RNAi genes in the conidia and mycelia of the highly virulent isolate T2 and less virulent isolate Rec; the results are shown as fold-changes including standard deviation and statistical significance, where the expression of each gene was statistically analyzed in four conditions: T2 XSM mycelia and conidia and in Rec XSM mycelia and conidia. Same letter samples are not significantly different.

| Gene | *V. nonalfalfae* material | | | |
| --- | --- | --- | --- | --- |
|  | T2 XSM mycelia | T2 conidia | Rec XSM mycelia | Rec conidia |
| VnaAGO1 | 8.4 ± 1.1 c | -2.2 ± 0.1 a | -1.1 ± 0.4 b | -1.5 ± 0.02 b |
| VnaAGO2 | 5.4 ± 0.9 f | -1.5 ± 0.01 d | -2.2 ± 0.1 e | -1.4 ± 0.1 e |
| VnaDCL1 | 2.2 ± 0.6 i | -2.3 ± 0.1 g | -3.0 ± 1.1 h | -8.4 ± 1.1 g |
| VnaDCL2 | 2.1 ± 0.4 l | -1.1 ± 0.1 k | -1.1 ± 0.1 l | -3.4 ± 0.2 j |
| VnaRdRP1 | 17.2 ± 0.9 o | -2.2 ± 0.04 m | 1.2 ± 1.1 n | -1.7 ± 0.02 n |
| VnaRdRP2 | 3.4 ± 0.6 s | -2.5 ± 0.02 p | 1.0 ± 0.4 r | -6.1 ± 0.02 p |

Supplementary Table 2. Expression levels of the *V. nonalfalfae* RNAi genes in the *V. nonalfalfae*-infected hop roots and stems of the susceptible Celeia and resistant Wye Target cultivars; the results are shown as fold-changes including standard deviation and statistical significance, where the expression of each gene was statistically analyzed in four conditions: Celeia roots and stems and in Wye Target roots and stems. Same letter samples are not significantly different.

| Gene | *V. nonalfalfae*-infected hop material | | | |
| --- | --- | --- | --- | --- |
|  | Celeia roots | Celeia stems | Wye Target roots | Wye Target stems |
| VnaAGO1 | 0 | 30.1 ± 1.2 b | 9.6 ± 0.4 a | 0 |
| VnaAGO2 | -1.0 ± 0.2 c | 3.4 ± 0.7 d | 1.1 ± 0.1 c | 2.5 ± 0.3 d |
| VnaDCL1 | 2.1 ± 0.3 e | 7.3 ± 0.5 f | 2.6 ± 0.1 e | 0 |
| VnaDCL2 | 1.2 ± 0.1 g | 4.4 ± 1.0 h | 1.3 ± 0.3 e | 10.3 ± 0.1 i |
| VnaRdRP1 | 3.1 ± 0.2 j | 6.3 ± 1.1 j | 2.8 ± 0.2 j | 34.0 ± 0.6 k |
| VnaRdRP2 | 5.1 ± 0.5 l | 14.3 ± 0.5 m | 6.7 ± 0.2 l | 0 |

Supplementary Table 3. Primers used in the RT-qPCR analysis of the fungal and *V. nonalfalfae*-infected hop materials.

| Gene | Forward primer | Sequence | Reverse primer | Sequence |
| --- | --- | --- | --- | --- |
| VnaAGO1 | AGO1A_F | CCCATCTGGATGCTTATCTCA | AGO1A_R | GTTCATGCATTCAAGCACCTT |
| VnaAGO2 | AGO2B_F | TCTGCCATCCGTGGATACTTT | AGO2B_R | CGAAACGGCAGTCCTGAAGA |
| VnaDCL1 | DCL1C_F | CGAAACAACCCGTGGTAGAAG | DCL1C_R | TAGCGGCTGGAGAATCACATAA |
| VnaDCL2 | DCL2B_F | TCATTCTAGCTGTGCGAACCT | DCL2B_R | CCACTGCCTGTGTCCATCTAT |
| VnaRdRP1 | RDRP1A_F | GAGAGTCATGAAGCCAACTGC | RDRP1A_R | ACGACAACTTTTCGGACAATG |
| VnaRdRP2 | RDRP2A_F | CGACAAACCACTATCAAAGCATCA | RDRP2A_R | TGGTGGTTTCCGAGTTCTGAA |
